# Diverse coping strategies for food insecurity: A qualitative study of economically precarious households in India in the context of COVID-19

**DOI:** 10.1371/journal.pone.0350020

**Published:** 2026-06-10

**Authors:** Charumita Vasudev, Swayamshree Mishra, Ankita Rathi, Jasmine Fledderjohann, Sukumar Vellakkal

**Affiliations:** 1 Faculty of Social Sciences, Lancaster University, Lancaster, Lancashire, United Kingdom; 2 Department of Economics, Indian Institute of Technology, Kanpur, Uttar Pradesh, India; Independent Global Health Consultant, INDIA

## Abstract

**Objective:**

The study examines how households in two Indian states managed food insecurity in the context of COVID-19, focusing on differences between migrant and non-migrant households in rural and urban areas.

**Methods:**

We integrate Davies’ framework of coping strategies with the Sustainable Livelihoods and Resilience frameworks to analyze how coping capacity is shaped by structural inequalities, existing resources, social networks, and access to entitlements. Between December 2022 and March 2023, we conducted 343 semi-structured interviews in 86 households in Uttar Pradesh and Goa, purposively sampled by migration status, location, caste, and household type. Thematic analysis was complemented with narrative analysis of 60 interviews from 15 households that had experienced severe COVID-related shocks, including job loss, reverse migration, and illness.

**Findings:**

Strategies ranged from routine adjustments—dietary substitutions, portion control, and pooling resources—to erosive responses such as maternal buffering, selling assets, accumulating debt, withdrawing children from school, and delaying healthcare. Rural non-migrants drew on kinship ties and PDS support, while migrants, especially circular migrant workers/recent arrivals were excluded from both entitlements and social support networks. Reverse migration reconnected households with rural networks but also strained agrarian systems.

**Conclusion:**

Migration status and rural–urban location critically shaped resilience. Coping responses to COVID-19 depended less on income loss than on structural access to entitlements and social support networks, highlighting the need for inclusive, context-specific, and targeted social protection.

## Introduction

Although government policies to address food insecurity in India, such as the Mid-day Meal Scheme, the National Food Security Act, and Poshan 2.0, have had some success, hunger and undernourishment remain persistent. The Global Hunger Index 2024 [1] ranked India 105 among 127 countries, labeling hunger levels as ‘serious’ and around 13.7% of the total population as undernourished.

Against this backdrop, disrupted livelihoods, significant health shocks, and unprecedented challenges in global food supply chains during the COVID-19 pandemic have raised serious concerns about the loss of progress in meeting nutrition-related Sustainable Development Goals by 2030 [2,3]. Close to 4.1 million youth in India lost jobs during the pandemic [4]. The worst hit were approximately 200 million circular migrants—migrants who have a weak or no foothold in the destination [5] and exhibit both short-term and long-term patterns of return to places of origination [6]. COVID sparked reverse migration to rural areas, creating intense pressure on rural economies and worsening inequality [5,7]. Although lockdowns have long since ended, as have most COVID-specific government and non-governmental support programs, there are lingering impacts of the pandemic [5,7].

Our paper examines household food management strategies during and after lockdowns to understand the pressures families already living in precarious conditions faced in relation to COVID. We situate our analysis within sustainable livelihoods [8] and resilience frameworks [9–11] to examine how the political economy of rural livelihoods, embedded in global processes, dynamic markets, and structural power relations, influences households’ resilience and adaptability and, consequently, their coping responses. We further use this integrated conceptual framework to explore both short-term changes to food practices and the longer-term impact of income disruptions on both migrant and non-migrant households who live in chronic precarity to understand how resilience is shaped not only by access to resources but is also cultural, context-specific, and shaped by access to social capital [10].

### Conceptual framework

Resilience entails the *capacity* of individuals, households, or community members to adapt, to change, to anticipate, or to respond to shocks and uncertainty [12], recognizing their agency and ability to make informed decisions that impact their life [11]. Scholars [9] argue that resilience results from interactions (synergies and trade-offs) between three capacities*— absorptive, adaptive and transformative—*each of them leading to persistence, incremental adjustments or transformational responses, respectively. However, this is not a simplistic linear response—absorbing low-intensity shocks, adapting during periods of greater disturbance, and transforming when conditions become unbearable. Instead, the resilience approach takes a systemic view of socio-ecological interactions by situating vulnerability (the susceptibility to harm) within multiple interacting stressors and power hierarchies, operating simultaneously at various levels and impacting individuals and communities unequally [11]—thereby shaping individual and collective abilities to absorb/adapt to shocks.

Resilience is thus best understood as a characteristic that can be either supportive or erosive, depending on the strategies adopted and their consequences [11]. For example, individuals can experience severe poverty but appear resilient by absorbing/adapting to poverty and deprivation by making choices that compromise their well-being— a concept referred to as *adaptive preference*. This occurs when individuals living in precarious situations adjust their expectations and aspirations to cope with adverse realities through detrimental practices such as skipping meals, withdrawing children from school, or delaying healthcare. From a social-ecological perspective [9], a crucial aspect of resilience is ‘*precariousness’* or closeness of a system to a *no-recovery threshold*, meaning that adaptive preference might be the only choice in times of crisis when households or communities routinely operate near threshold limits in everyday life.

In this context, to understand the coping responses of households to COVID-19, we use the Sustainable Livelihoods Framework [8](SLF) to situate the households in the broader context of socio-historical origins of structural inequalities and the recent global/local shifts that have compounded existing/created new vulnerabilities.

In India, the onset of neoliberal globalization has spurred greater integration of local economies with national and global circuits of capital accumulation. While this has led to increased aggregate incomes and more economic opportunities for some, it has also created newer forms of rural dispossession and exacerbated livelihood insecurity for marginalized households [8]. The deepening of agrarian capitalism caused by neoliberal policies has increased the casualization of labor, pushing the rural poor to sustain themselves through spatially fragmented and precarious wage work in both agriculture and non-farm sectors [13]. Declining rural and farm incomes, coupled with climate-induced shocks, have forced families to sustain themselves through irregular, precarious work and cyclical loans for agricultural inputs—often making food security and everyday life contingent on loan repayment cycles. More integrated economies have triggered migration outside of the villages, not only reshaping daily interactions in the village but also spatially distributing risks by creating multi-sited, trans-local households that are sustained through remittance networks and affective ties [8].

Rooted within this dynamic context of adverse incorporation [8], wherein poverty is systemically created and maintained through the incorporation of the working poor into markets on adverse terms, are existing social hierarchies or ‘relational power’. In India, these include structures of caste, class, and gender, which shape unequal access to resources, opportunities, and social capital. From a political economy perspective, this relational power is itself embedded in historical access to labor markets, social protection systems, and crucial environmental resources, including agricultural land—the uneven distribution of which structures differential vulnerability and resilience to shocks. Neoliberalism thus amplifies existing social hierarchies, as those with limited access to land, capital, and social networks are systematically excluded from access to socio-economic opportunities and support, and thus disproportionately face the consequences of shocks. For example, the caste-class nexus intersects with the lack of local social support networks to limit the capacity of circular migrant workers who survive on informal daily-wage work to respond to shocks.

**Fig 1:**
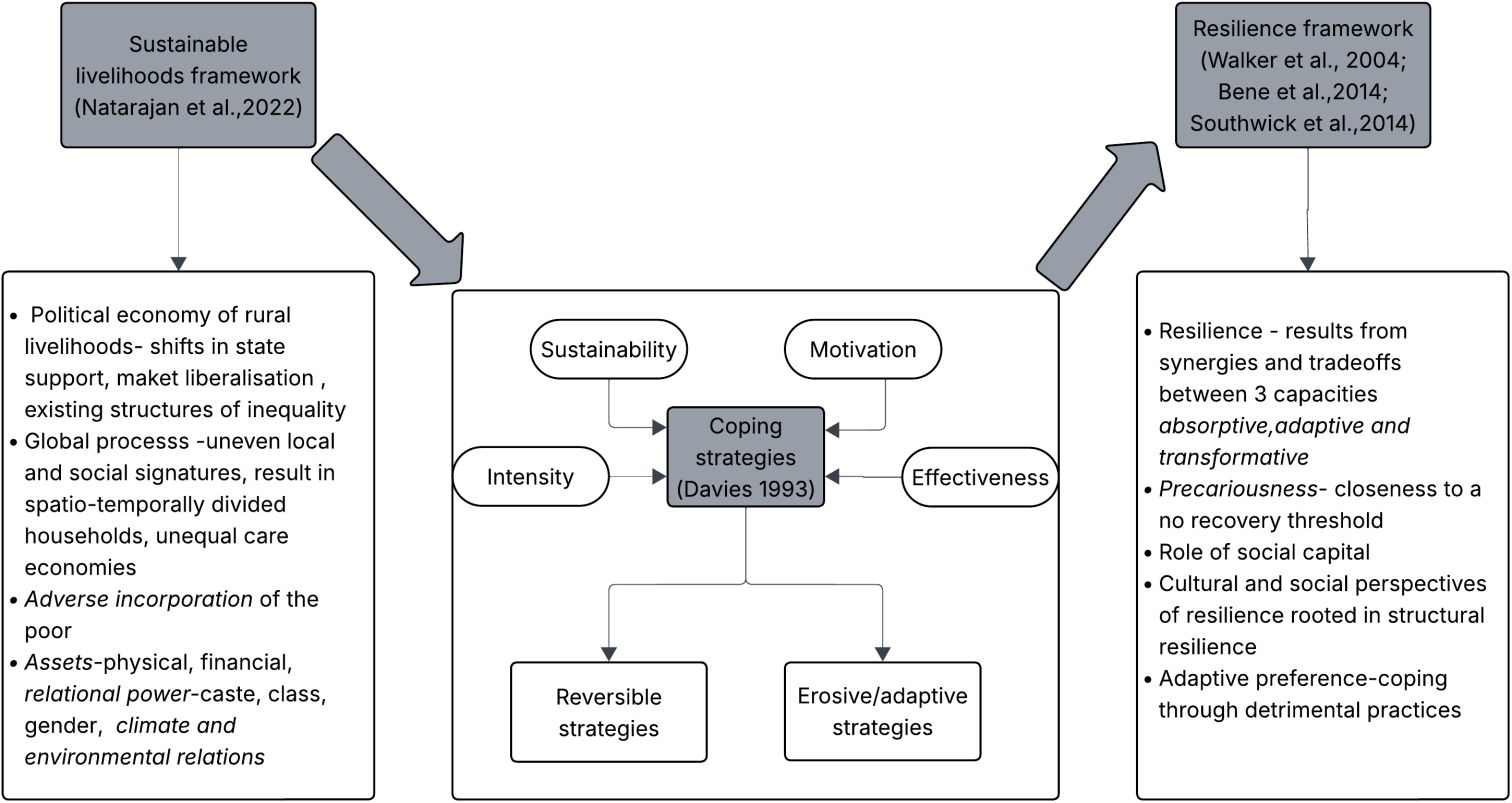
Integrated Conceptual framework.

In this integrated context of understanding resilience as situated within the SLF framework, we use the typology of Davies’ coping strategies [14] to examine how households managed their basic need for food in the context of COVID-19 (Fig 1). To categorize strategies as either *short-term/reversible* coping strategies or *adaptive* strategies, we use Davies’ critical conception of coping strategies as informed by the *intensity* of their use (how dependent the households are on these strategies compared to ‘normal’ times), the *sustainability* of these strategies when intensity increases, the *motivation* behind their adoption, and their *effectiveness* in meeting food needs. If adaptive strategies adversely affect future coping capacity, they are categorized as *erosive*.

This integrated framework illustrates how the coping strategies households employ are situated in existing ‘precariousness’ —that is, not only shaped by socio-historical processes and local/global market forces but also reinforced through structures of inequality like caste, class, and gender—that mediate unequal access to resources, social support, and entitlements [10]. Through this lens, a major global disruption like COVID produced novel, unequal outcomes, but also exacerbated existing vulnerabilities and inequalities. Disasters such as COVID-19 are not ‘natural’ shocks to an otherwise well-functioning system; they are, instead, a high-visibility indicator of interconnected and longstanding inequalities that created the conditions for systemic marginalization [15].

Building from this conceptual framework, our analysis is motivated by the following questions about households’ experiences and coping strategies during COVID-19:

- What strategies did households adopt to manage food?
- How did these strategies utilize changes to government entitlements?
- How were strategies influenced by geographical and socio-cultural location?

## Methods

We use primary semi-structured interview data collected over three months, supplemented with household roster and observational data, to understand how various household members experience food insecurity. The project’s field sites are in two Indian states: Uttar Pradesh (UP) and Goa.

### Field sites and sample selection

UP is a north Indian state with a population of more than 241 million. It is primarily agrarian, with 65.5% of the population residing in rural areas, and has a higher-than-average poverty rate (39.8% compared to the national average of 29.5%). Only 10.8% of workers are engaged in regular or salaried work. The female workforce participation was particularly low (17%). While most of the population is Hindu (78%), a sizeable minority is Muslim (20%). We selected four districts (Kanpur Nagar, Kanpur Dehat, Unnao, and Kannauj) to maximize sample diversity and to cover both rural and urban areas. Selecting neighbouring districts reduced travel time and costs, allowing us to spend more time in the field.

In rural areas, the families in our sample were mostly engaged in agricultural labor, relying on seasonal crops, subsistence horticulture, and sometimes livestock farming. Children as young as 8–9 years were engaged in agricultural labor. Housing ranged from sturdy constructions with staircases and courtyards to makeshift shelters with rudimentary roofing. In urban areas, participants included both first-generation migrants seeking livelihood alternatives amidst agricultural challenges and long-term residents. Most of these households relied on contractual/daily wage labor and faced financial instability exacerbated by illnesses, marriage expenses, seasonal changes, and so on. Housing infrastructure and living conditions posed significant risks, with families of 4–5 individuals residing in single-room dwellings lacking basic amenities, including sanitation and electricity. We conducted 238 interviews in 59 households.

In UP, our sample includes two types of migrants: out-migrating individual male workers from rural to urban areas (whose families we have interviewed) and incoming migrant families (in both rural and urban areas). Individual workers were often circular/seasonal migrants, with few connections to the destination. These were also the migrant workers who returned to rural areas due to job losses and a lack of local support networks during COVID-19. Migrant families, on the other hand, are those who have moved to UP from neighboring states like Bihar.

In contrast to UP, Goa is a small coastal state in southwestern India, with the highest GDP per capita in the country and high literacy rates (88%). Manufacturing constitutes more than 40% of the state’s GDP [16] and tourism plays a significant role in the economy. We focused on migrant populations, who experienced heightened levels of precarity. According to the 2011 Census [17], around 43% of inter-state migrants to Goa originated from Karnataka, 26% from Maharashtra, and 12% from the states of Uttar Pradesh, Bihar, and Jharkhand, while only 6.7% came from southern states such as Kerala, Andhra Pradesh, and Tamil Nadu. We interviewed households in urban and peri-urban South Goa, including short- and long-term migrants from states such as Karnataka, Maharashtra, Bihar, Odisha, and Assam. In total, 105 participants from 27 households were interviewed.

In Goa, however, only incoming families have been interviewed (with only one family having a seasonally migrating male worker). These families comprise both first-generation migrants who moved to Goa themselves in search of work and established families here, as well as second-generation migrants (whose parents moved to Goa). They have varying degrees of enduring connections to their places of origin, including both affective ties and financial support from relatives in their home states.

From the full sample of 86 households, the study purposively selected 15 households that had experienced severe COVID-related shocks for in-depth narrative analysis, across both states. Detailed information about each of the 15 households in our sub-sample has been included in a supporting information file (S1 File_Description of sub-sample). The serial numbers in the supporting information file have also been referenced throughout the manuscript, along with narratives from each household. Additionally, any instance where a serial number is not provided indicates that the excerpt is not from the sub-sample.

We used purposive sampling to select households, maximizing diversity across caste, religion, location (rural/urban), household structure (single vs. multifamily), and family type (nuclear/single parent/multigenerational). (Details in Table 1.) For the sub-sample, too, we purposively sought to maximize diversity, focusing on including diverse experiences of COVID-related shocks across migration status (migrant/non-migrant), location (rural/urban), and household structure.

**Table 1:** Socio-demographic characteristics of the sample.

Variable		Total households	Percent	Sub Sample (Narrative analysis)
**State**		86		15
	UP	59	69%	9
	Goa	27	31%	6
**Location**		86		15
	Rural	33	38%	6
	Urban	53	62%	9
**Religion**		86		15
	Hindu	67	78%	13
	Muslim	16	19%	1
	Christian	2	2%	1
	Other	1	1%	0
**Caste**		86		15
	General	20	23%	3
	Other Backward Classes	24	28%	2
	Scheduled Caste	24	28%	5
	Undeclared	9	10%	2
	Not enough data	9	10%	3
**Household Type**		86		15
	Single household	72	84%	9
	Joint household	14	16%	6
**Family Type**		86		15
	Single parent	5	6%	1
	Nuclear Family	51	59%	9
	Multi-generational	28	33%	5
	Other	2	2%	0

Selection continued until narrative patterns reached thematic saturation.

### Data collection

The Faculty of Social Science Ethics Review Committee at Lancaster University, United Kingdom approved our study (FASSLUMS-2022–0953-RECR-3) on 10/11/2022. The study was also approved by Institutional Ethics Committee at Indian Institute of Technology, Kanpur (IITK/IEC/2022–23/I/19), the project’s host institution in India on 09/09/2022.

The fieldwork for this research was conducted between 22 December 2022 and 23 March 2023 by SM, CV, and AR. We collected basic sociodemographic details using a household roster adapted by JF from the Demographic and Health Surveys (DHS). This included questions about the age, caste, religion, education, and occupational status of each household member, as well as general questions about the dwelling infrastructure, sources of drinking water, sanitation, sources of fuel, etc.

After completing the roster with one adult member, we conducted semi-structured interviews in Hindi with multiple members of each household, including as many members aged 7+ as possible. We included children based on evidence that children’s life trajectories are significantly altered by food insecurity, yet older children-adolescents in particular are frequently overlooked in food insecurity studies. [18,19]

Recognizing varied levels of literacy among our participants and the possible stigma associated with limited literacy, in addition to sharing written participant information sheets (PIS), our team (CV, SM, AR) provided a detailed oral summary of their contents to all participants. We explained the objectives of the research, why participants were being approached, and the possible benefits and harms of participation. Additionally, the purpose of the audio recordings was explained to participants (both children and adults), and they were interviewed only if they were comfortable with the recording. Once the participants consented to the interview and audio recording, the recorder was started, noting the date, time, location, and household number of the interviewee on record, and noting the consent in simultaneous field notes. Because we never undertook lone work in the field, this was witnessed by at least one other team member. For children, parental consent was obtained, and then children’s assent was obtained in front of their parents. This procedure was approved by the ethics review boards of both the UK and Indian universities involved in the study. Refusal was acknowledged in various forms throughout the interview, whether verbally or nonverbally, at the start, through skipping a question, or by refusing to answer, even after consenting to the interview itself. The team’s contact numbers, and the heads of their departments were shared, and participants were informed that they could ask for their data to be removed even after participation.

Although we originally did not record conversations while completing rosters (assuming only ‘factual’ details would be shared, which would be documented in the roster), we began recording roster discussions when we realized that the roster sparked rich conversations as well.

We deployed age-specific interview guides (for ages 7–12, 13–17, and 18 + years). These were conceptualized in English by the project lead, JF, with inputs from the Co-I (SV) and project partners, then discussed and revised with inputs from the field team (CV, SM, AR), and collaboratively translated into Hindi by the field team. Questions on meal preparation and consumption, food preferences, and household inequalities were common to all instruments (wording varied somewhat by age group). The children’s guides had unique questions about schooling, meals at school, care work/responsibilities at home, and allocation of their preferred foods at home. Drawing was used as an (optional) strategy to help children open up and discuss food-related issues. We obtained children’s oral assent before photographing their drawings. The adult questionnaire had more questions about livelihoods, food prices and availability, retrospective reflections on COVID and its impact, social protection, climate change, and agricultural practices. We also included a version of the UN Food and Agricultural Organization’s Food Insecurity Experience Scale (FIES) [20] for adults and a modified version for children [21]. We modified the FIES by adding open-ended probes (e.g., ‘Can you tell me more about when that happened?’) to deepen understanding of food insecurity experiences. A summary of core questions in the household roster and the adult and children interview guides has been added as a supporting information file (S2 File_Core questions).

We piloted the interview guides in seven households in UP in early December 2022, including all eligible age groups, and then revised them to thematically cluster questions and avoid repetition. We prepared a small grocery basket (₹800) for each household to express our gratitude to the participants for their time.

We approached both interviewing and data analysis with continuous reflexivity [22], acknowledging how interviewer positionalities can affect participant sharing, interviewer responses, and other interpersonal dynamics. We (CV, SM, AR) are three female researchers born into Hindu families. We are all experienced qualitative researchers with prior experience in collecting data on sensitive issues in various locations in India. All of us have completed our doctorates in social science disciplines. When interacting with participants, we answered queries about the project and ourselves, scheduled interviews at the participants’ convenience, and tried to ensure as much privacy as possible while interviewing. In addition, we made continuous attempts to enable comfortable sharing among participants through regular check-ins and by repeatedly emphasizing that we were trying to learn from their lived experiences; thus, all their experiences were of value.

Audio recordings were translated into English, including pauses, false starts, laughter, and the associated background information/noise. Transcription and translation were carried out by the field team in discussion with JF, and by one additional translator from UP with a social science background, supervised by the field team. The field team also created a translation guideline document and a glossary of terms to be retained in Hindi when an exact translation seemed difficult.

### Data analysis

Data analysis was conducted in two stages. First, we conducted a broad thematic analysis by carefully familiarizing ourselves with the data, coding, and reviewing codes for interviews from the entire sample. These were then differentiated by rural-urban location and migrant/non-migrant status to understand their diverse experiences of COVID. Drawing from the above integrated framework, we categorized results into three broad themes: 1) Strategies for food management, 2) Government support easing resource constraints, and 3) Rural-urban linkages. While the first theme keeps its focus centered on the households to understand coping strategies deployed during and after COVID, the second and third themes take a step back and explore the local, national, and global processes, social policies, and structural inequalities that limit a household’s choice of coping strategies and subsequent resilience for such future events.

To grasp the nuances of the strategies households adopt, we delved further into our intrahousehold data to identify diverse experiences within households, some of which were spatially fragmented. In this stage, we paired the thematic analysis with a narrative analysis of 60 interviews with members of 15 households (details in Table 1 and S1 File_Description of sub-sample) that we identified through our process of data familiarization and coding as having been especially impacted by COVID. These included households where members had either died due to COVID/related illnesses or those which faced lingering impacts of untreated illnesses that occurred during COVID; households where additional family members had to be supported due to reverse migration; and families who had not recovered from economic shocks posed by loss of a job/business during COVID. This added layer of analysis was intended to further illustrate the nuances in a household’s response to an external shock by studying the interviews of all household members in tandem. Narratives of each generation reflect a social and historical location from which an individual constructs and articulates their lived reality [23]. Thus, to understand the meanings different members of households attached to food practices as a response to the additive constraints posed by COVID, interviews with all members of one household were examined simultaneously, paying attention to the three-dimensional aspects of sociality, temporality, and physical space [24]. In the case of our transcripts, this meant paying attention to how the COVID stories were shaped not only by individuals’ socio-cultural and geographical locations but also by their positions within the household and the continuity of events/relations before and after the lockdown period.

Transcripts were coded in NVivo 12 and supplemented with additional field notes on the data collection sites and nonverbal cues observed by the interviewers. The coding process was primarily inductive. Initial codes were identified through data familiarization. These were then grouped into relevant themes to improve analytical coherence in interpretation. For example, all food management strategies like reducing consumption, buying less quantity, buying cheaper quality, avoiding certain food items, adults managing by reducing self-consumption, managing food budgets, pooling resources, etc., were clubbed under three sub-heads: 1) Consumption cuts, 2) Changes to household composition, and 3) Tough Trade-offs. A quantitative summary of a few themes/associated codes is available as supporting information (see S3 File_Quantitative summary of themes). For the narrative analysis, we examined interviews from each household together to understand the meanings attached to each decision and its impacts, as shared by all household members.

### Inclusivity in global research

Additional information regarding the ethical, cultural, and scientific considerations specific to inclusivity in global research is included in the Supporting Information (S4 File)

## Results

Using the integrated framework, we discuss how resilience is shaped by migrant status, rural-urban location and existing structural inequalities. These differences shape access to physical and financial resources, social capital or ‘relational power’ [8] and government support. We find that for households that were already experiencing substantial economic precarity and operating close to the resilience threshold, access to social networks, agricultural food stocks, and government entitlements were critical for food-related coping.

Drawing from these compounded support structures/lack thereof, households adopted coping strategies, some of which can be classified as reversible/short-term, such as reducing food diversity or switching to cheaper alternatives (non-erosive), while others had longer-term repercussions adversely affecting households’ future ability to cope or their mobility goals (erosive), such as sale of assets or withdrawal of children from school or delaying healthcare expenditures. For some, asset ownership and availability of social support networks, which are often rooted in hierarchies of caste, class, and gender, provided greater access to adaptive strategies like pooling resources and accessing local support, delaying the need for erosive coping. Public entitlements also temporarily shifted households away from resilience thresholds, providing support to manage limited resources. We also note that, for some households, coping strategies such as reducing the quality and quantity of food were longstanding and were exacerbated by COVID, rather than being novel strategies.

**Fig 2:**
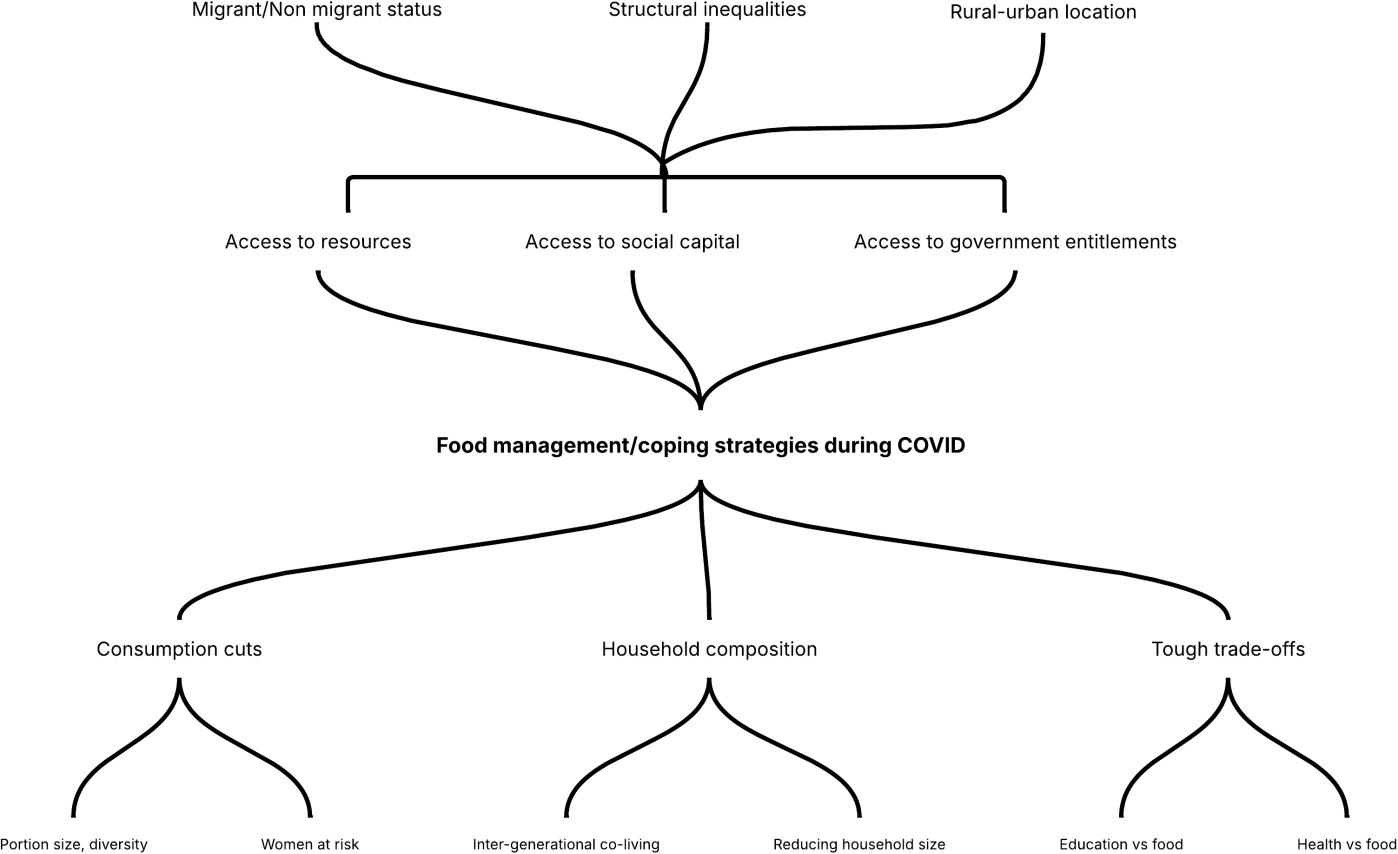
Food management strategies in the context of COVID.

Fig 2 summarizes the various themes discussed in the next section.

### Food management strategies

#### Drastic consumption cuts.

As employment became irregular, households’ first strategies were to smooth consumption by shifting to less preferred foods, reducing expensive items like dairy and meat, and limiting portion sizes. However, what constitutes a ‘routine’ response versus a shock coping strategy is also based on socio-cultural contexts and existing vulnerabilities. For example, what might be a shock-coping strategy for one household might be a routine coping strategy for a particularly marginalized household.

All families in our sample reported compromising dietary diversity during the lockdown period owing to both accessibility and affordability constraints, with potatoes and cereals becoming primary fallback options. In precarious households that regularly experience seasonal disruptions in livelihoods, these strategies are often used as short-term absorptive mechanisms in times of stress. However, as lockdowns further compromised livelihoods, more severe measures such as borrowing for food, skipping meals, and reverse migration to rural areas were adopted.

Davies [14] distinguishes between routine adjustments in sensitive livelihood systems and coping strategies adopted in times of shock. We argue that among households surviving close to resilience thresholds with limited access to social networks and government support to fall back on, routine coping might compulsively have to give way to erosive coping strategies during shocks, or adaptive preference [11]—affecting the existing and future coping capacities of the socio-economic systems they are situated in. While limiting food diversity, controlling portion sizes, or eating less nutritious meals are routine strategies in certain periods of the agrarian cycle in some areas (short-term absorptive/adaptive strategies), more severe adjustments like skipping meals, going hungry for (an) entire day/s (disproportionately women), and debt-financed consumption are structurally constrained coping responses to disruptive events like COVID-19 and can be erosive.

Locals in both rural and urban areas benefited from social connections established over generations and drew on these long-term local networks to manage day-to-day expenses when income became irregular. More recent migrants to urban areas, by contrast, rarely had access to these connections, which are often caste, religion, or neighborhood-based. The return of single male migrant workers to origin towns in UP, often in adverse circumstances, speaks to a lack of fallback options/local support structures to survive without daily wages. This further links back to structural disadvantages in asset ownership rooted in caste-class hierarchies, along with adverse incorporation during neoliberal restructuring that had produced rural dispossession necessitating out-migration, only to be followed by a similar lack of support and protection at the destination.

Additionally, as Palma and Araos [25] argue, some households are more resilient or better equipped than others to manage a crisis, not only based on their socio-economic situation but also on their ability to understand external threats and mobilize resources. For example, some longer-duration migrants benefitted from local relationships of trust and could negotiate delayed payments for essentials. One multi-sited family from Odisha (S.no. 12, urban migrant household), living in Goa for around 10 years, bought their groceries from a local shopkeeper who was also originally from Odisha. They accumulated loans of ₹20,000 during the lockdown period, which they were slowly repaying. Conversely, some locals faced greater adversity due to neighborhood or family dynamics. For example, in rural UP, a lower-caste family described how their concerns, such as water supply, tube well repair, and road maintenance, often took longer to address because they lived in an upper-caste neighborhood and the village head was also upper-caste. Similarly, a 38-year-old Hindu migrant woman in Goa shared that they faced difficulties because her husband did not contribute anything to the household (even though he was a skilled worker), so she had to manage everything alone for her four children aged 8,7, 6, and 5 years. Thus, absorptive and adaptive capacities were determined by a complex interplay of intersecting identities and related access to local support structures. Similarly, for those without access to local support networks, relatively smaller risks/crisis could lead to erosive coping.

In both local and migrant families, women, especially mothers, frequently enacted maternal buffering [26], that is, compromising their own food and nutrition to manage food for the family when resources become scarce. While talking of COVID, women reported surviving on salt water/sugared water for 2–3 days or eating only salt-roti so that children could be fed and men could continue working/searching for jobs. Despite this, in several interviews, children reported sleeping ‘just like that’ (hungry). Coping strategies of women within households were thus rooted not only in structures of gender but also in intersectional identities that shape normative ideals of femininity/motherhood, influencing their daily negotiations with food and their decision to de-prioritize themselves to feed other family members better. The consequent nutritional deprivation resulting from such choices can have adverse impacts on women’s health and erode future resilience of households.

### Adjusting household composition to make ends meet

Another way households cope with crises is by changing their *household composition*. This could be achieved through household extension via intergenerational co-residence [27] or by sending children away to live with relatives, or by some members moving away to reduce household size [28]. This could be considered an adaptive measure in principle, as it involves a structural change in household composition. However, it had limited sustainability in the context of COVID-19, as compounded stressors like increased medical expenditures and loss of income further exacerbated existing vulnerabilities.

In some families, household extension meant that those previously operating as nuclear units for daily expenses began sharing resources, such as cooking fuel and electricity costs, by jointly preparing meals with relatives living nearby. This temporary shift towards resource sharing reverted to the nuclear model once employment stabilized after the lockdown.

**Fig 3:**
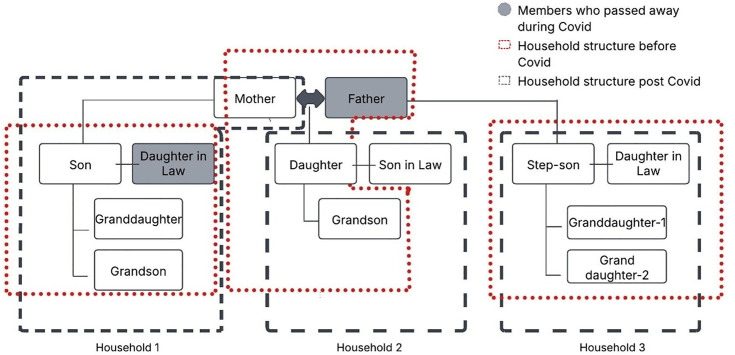
Changes to household composition during and after COVID-19 lockdown.

In one such case (Fig 3), three distinct households, comprising members of the same extended family, occupied separate rooms within the same premises both before and after the lockdown. However, the pandemic significantly altered their intra-household dynamics. The daughter (household 2) noted that the families began cooking together to conserve cooking fuel and to ensure that the children did not go hungry. Despite these coping mechanisms, the household was unable to mobilize sufficient resources when her father fell ill, leading to his death during the pandemic. Drawing from Davies’ [14] while pooling resources might have been an effective coping strategy with respect to food, its *sustainability* became limited as the intensity of shock increased, compounded in this case by health expenditures. During the interview, she became emotional as she recounted how, despite her best efforts, she felt she had failed to provide adequate care. She said,

*He became paralyzed later and during COVID...his condition was so bad that he was ill, and he was not taken to the hospital… I used to sell masala (betel nut mixture) all day… and throughout the day, I used to think I should collect enough money before I go home and bring painkillers to my father… feed him fruits etc. so that he becomes well as before…My father was very good. He never let me feel any dearth of anything… like shade from the sun.*

(S.no 2.,Woman, 38, urban-non-migrant, Scheduled Caste)

The figure denotes how the composition of three households residing in one premises changed due to COVID-related events and scarcity. The red dotted lines denote each household’s composition before COVID-19 and grey dashed lines denote the household composition of each household when the fieldwork was done (March,2023).

A disabled single mother, she recounted that she remarried during COVID, against her mother’s wishes, hoping for financial stability. Her ten-year-old son remembered the period as particularly difficult, noting that his stepfather was unable to secure employment, which left the family struggling to meet basic food needs. He explained that he sometimes lied to his grandmother that food was cooked in their nuclear household, so she would not worry.

*At that time, papa did not have work... so he used to do small work here and there and like… if he got 500 rupees, then that money would run the house…Like sometimes the food was not made… if there is only a little flour in the house, then it used to be made and eaten.*

(S.no 2, Male child, 10, urban-non-migrant family, Scheduled Caste)

In Household 1, the daughter-in-law passed away shortly after the father’s death. Her husband (Son, Household 1) was absent from childcare when she was alive; her passing left the children without material or emotional support. Their grandmother (Mother, Fig 3) accepted care responsibilities and used her contacts to obtain a government loan for a small shop to generate income. By our fieldwork in February 2023, these families returned to a ‘new normal’ with three separate households again, although quite different in structure from pre-COVID times. However, the earning members asserted that livelihood opportunities had not returned to pre-COVID levels.

In a migrant household in Goa (S.no. 11, urban-migrant family from Karnataka, Scheduled Caste), the eldest of the four children grew up with the husband’s financially better-off parents to ease the financial burden on their nuclear family. During COVID, another daughter was sent to live with the grandparents when managing finances became difficult. Thus, while livelihoods had limited adaptability due to structural constraints, it can be argued that such adaptations made by families helped to manage resilience by temporarily shifting households away from threshold limits [9] and ensuring that long-term sustainability was not affected.

However, for individual migrant workers, changes to household composition upon their return put additional strain on the already limited resources. Distress-motivated reverse migration during COVID lockdowns led to unexpected changes in household composition, especially in rural areas. Rural-to-urban migrants in UP tried to move back to their villages, assuming that managing food would not be an issue, given access to agricultural stocks and/or social networks. In a joint family in UP, two unmarried brothers returned from working in the city to the rural area where their eldest brother lived with his wife, four children, and an unmarried sister. The married brother (MB) and his wife noted that it became tougher to provide for their children with additional people in the household.

*MB: Like during COVID, everything was tight. Now the brothers who lived away…there was no work for them, so they also came here.*

*Interviewer: So you had to manage for everyone?*

*MB: Of course, whatever food is there, the entire family will eat... See, this is the problem, if there are two earners then there is more money, if there is a single earner, then the money will be tight.*

*Interviewer: So how was it managed during that time brother?*

*MB: With god’s grace, everything went on. And then it is the government. They gave ration etc.…they helped a lot during that time.*

(S.no 5, Man, 55, rural, non-migrant, general caste)

The unmarried brothers in the household were also worried about having limited opportunities to return to the city or to find local work. Speaking closely to the idea of their household operating near the boundary of a resilience threshold, the unmarried sister jokingly used a local idiom to compare their household’s situation during lockdowns to a faulty vehicle that must be manually pushed to start (‘*Dhakka plate gaadi’*), indicating that they had no choice but to push through tough times. All members remembered this as an extremely stressful arrangement, as work opportunities remained scarce.

### Tough trade-offs

Davies [14] warns that short-term coping strategies might have lasting consequences on long-term development. This occurs when a choice is forced between basic subsistence and long-term sustainability [29], leading to the adoption of strategies that are erosive in the long term and hamper future resilience [10]. An important reported example of this in the context of COVID was the choice between continuing children’s education and managing everyday food expenditures. We found that some children had not returned to school, as families struggled to regain pre-COVID income levels or were missing school for prolonged periods to augment family income. Intensification of wage work by increasing the number of earning members is an important routine coping strategy for poor households [30]. Numerous children talked about having had to leave/skip school, shift from private to public schools, or start agricultural labor to contribute to household earnings because of financial troubles after the lockdown. Som‌‌e families prioritized resources to educate their sons, while daughters were withdrawn.

In one household in Goa, the father’s small business became unsustainable during lockdown, and he went into hiding to avoid debt repayments, leaving his wife and three young children behind. The wife recalled struggling to find a place to stay, barely feeding the children by working as a house helper and taking informal loans. Although her daughter returned to school post-lockdown, she was grateful that schools were closed during the lockdown, as her older daughter could take on care responsibilities for her siblings.

*Good thing the school was closed. Everyone says it would be better if school was open, I pray it was better that the school was closed. Because the children were at home, I got to go for work. Otherwise, how would I have done this, …there was a small child, how could have I done work and all?... daughter used to take care of him, the elder daughter. Both the children.*

(S.no 15, Woman, 36, rural migrant family from Karnataka, Muslim)

In other families, people spoke of impossible choices during the pandemic between medical expenditures and managing food—the ‘treat or eat’ dilemma [31]. For example, a woman in Goa managed without medicine for her two-year-old’s high fever, since buying medicine would have meant that family members would go to bed hungry (S.no 13, urban migrant family from Madhya Pradesh). In another household from UP, a participant discussed how they had to eat plain roti when medical expenses cut into food budgets.

*Participant: Like if I have 200rs, I spent that on medicines,then I can’t buy vegetables, what will we do then, you tell? So that happens.*

*Interviewer: So, what is cooked that day?*

*Participant: Then nothing, just eat it dry (sookhi’- reference to wheat roti). If the entire money is spent on medicine so where will I get it (vegetables) from?*

*Interviewer: So, does everyone in the family eat dry then?*

*Participant: What else.*

(S.no 5, Woman, 43, rural, non-migrant, general caste)

Non-crucial medical expenses were deprioritized to make room for everyday expenses. In another migrant family in Goa, a father of four delayed operating on his back injury so his wife’s income could be prioritized for children’s expenses during the lockdown (S.no11, Man, 38, urban migrant from Karnataka, Scheduled Caste).

These trade-offs are often undesirable decisions of last resort (adaptive preferences) in precarious households when other social or state support is unavailable to absorb shocks, and they can have lasting consequences for future resilience by eroding earning potential and compromising families’ mobility goals.

#### State support easing resource constraints.

During COVID, adjustments were made to existing entitlements, such as the Public Distribution System (PDS), which provides state-subsidized cereals to eligible households, alongside several additional entitlements initiated by state and central governments to address heightened food insecurity during lockdowns. This helped households to allocate limited resources towards non-food expenditure and manage household expenses.

Pradhan Mantri Garib Kalyan Anna Yojana supplemented PDS during the pandemic by providing an additional 5 kg ration per person per month, doubling the usual PDS allocations and also making them cost-free. Participants reported that this was indispensable government support that helped them survive that period, temporarily moving these precarious households away from the threshold of resilience [9]. Many households relied solely on PDS grains, especially during the pandemic, with rice/roti/potatoes (if stored post-harvest) and salt as common meals during the lockdown. While the added central allocation has been withdrawn, UP has continued to provide free rations (Atma Nirbhar UP Free Ration Scheme) under the PDS to eligible households, as families still struggle to find sustainable livelihoods.

In addition to increasing the quantity during the pandemic, the government also diversified PDS allocations by including items like cooking oil, salt, and chickpeas in both UP and Goa. Numerous households reported not being able to afford these expensive items before or after the lockdown period. When asked if there was anything else the government could do to help families like theirs, a participant in UP (Male, 38, urban, non-migrant, Scheduled Caste) shared that they were really benefitting from generic medicine shops (Jan Aushadhi Kendra), suggesting a similar approach towards groceries, where subsidized, quality-monitored local staples could be sold to aid nutritional diversification of entitlements.

However, families without access to PDS allocations, specifically migrants who lacked local registrations, were especially vulnerable during this period. Migrant brick kiln workers (S.no 4, rural migrant from Bihar, caste not known), for example, explained that their PDS registrations were in Bihar, so allotments were inaccessible in UP. However, a hospital (in collaboration with a local NGO) provided two cooked meals daily for two months during the lockdown to these families in a colony of seasonal migrant kiln workers.

Some migrant families with registrations in their place of origin reported that other family members relied on those rations in their hometowns. Some had parents who were accessing their allocated rations in the village, or children who were reliant on them while staying with relatives. For example, a migrant family (S.no. 12 urban migrant from Odisha, caste not known) had left their oldest daughter with her maternal grandmother, who had been raising her since about 10 years. The grandmother and child accessed the family’s share of the rations in Odisha, which our participant saw as essential for her mother and daughter’s sustenance. Some migrant families living in Goa for as long as 10 years did not have local PDS registrations. While this can partly be explained by a lack of proper documentation and the nature of their jobs, which forced them to constantly move to wherever work was available in the short term, this also speaks to the functional multi-sitedness of households. While declining rural incomes and greater economic integration forced some family members to migrate to non-agricultural jobs in the hope of better daily wages, other vulnerable family members (often parents, young children, and women) had to be left behind. Families’ unwillingness to change PDS registrations to local ones, even after years of residence, often stems from these continuing affective ties and remittance networks. While this might be seen as an implementation gap that allows rations to be mis-targeted, in a local context, it is also a policy adaptation that enables families to manage food insecurity across complex, multi-sited family structures. Public support schemes like the PDS thus become especially crucial risk-management strategies for these multi-sited marginalized families who have limited/no access to assets and social-support networks and are thus reliant on their daily labor for sustenance—an outcome closely linked to neoliberal restructuring.

In Goa, a local lockdown modification of the Mid-Day Meal Scheme (providing free cooked meals in schools) involved sending dry rations to vulnerable families. In addition, the government provided temporary doorstep rations in some urban areas. In others, people with a labor card received a temporary monthly allowance. While PDS and additional government allocations were the main form of support in rural areas, local civil society organizations supplemented and sometimes filled an important gap in coverage by providing hot-cooked meals, doorstep rations to migrant families.

These additional measures especially helped migrant families who did not have access to PDS rations by smoothing food availability and coping capacity of households and thus temporarily mitigating the compulsions for adopting erosive coping.

#### Rural-urban linkages: Diverse yet shared vulnerabilities.

In rural areas, several households reported that agricultural/industrial labor still provided jobs (with delayed/erratic payments), especially given the lockdown exemptions for agriculture. In urban areas, it became impossible to find jobs, especially for the daily wage migrant workers, triggering a wave of reverse migration to rural areas.

However, within urban areas too, there were disparities among the laboring families based on the type of work and associated local social networks, which are strongly shaped by caste and class. While most informal laboring migrant families/workers in urban areas faced threat to their means of employment, a few non-migrant families continued to find work due to the association of their work with higher officials/those working in essential services. For instance, despite the widespread precarity experienced by many drivers in urban contexts during COVID, one participant interpreted his relative stability as being shaped by his caste and class position. He repeatedly stressed how his caste location enabled him to access more advantageous social contacts and maintain them. In this sense, both caste and class networks appeared to function as a safety net. When asked about his family’s experiences he shared:

*Child, for us, during Corona… there were no problems for us...I was working with a doctor; I did not even have a minute to spare. There was scarcity at labor level work,otherwise the adhikari (higher officials) had a lot of work. And my contact and work is with the adhikari (higher officials), so there was no question of having problems. I did not face any difficulty.*

(Male, 46, urban, non-migrant, general caste)

While the need to work amid risk to health and life is also reflective of how informal workers coped with crisis, we found that laboring families belonging to better-off castes and class were able to rely on their caste-based networks and long-term work affiliations to find work to cope with crisis. In a rural higher caste family (S.no.5), when two more brothers returned to the village, unable to find work at the destination, the local married brother who worked as a contractual employee in a factory was able to negotiate some limited days of work for both of his brothers with the owner, based on his long-term association. This was even though the factory was operating at reduced hours. This limited additional income was crucial in helping meet the expenses of the extended family. However, these networks of support were not equally available to all, as some lower-caste-class households, including rural non-migrant daily wage workers, struggled to find work.

In rural areas, ownership of assets, like land and cattle—often embedded in caste-class structures benefiting the most privileged in these hierarchies—also served as important cushions to navigate the crisis and manage food insecurity in the context of the shock produced by COVID. Some agrarian families in rural areas had enough cereal to eat (produce from their own land/return from crop-sharing arrangements) but were struggling to sell produce and consequently had limited/no disposable income. Similarly, while lentils were inaccessible to most families, some cultivating families had stored stocks but limited cash flow to buy other items. Some marginal farmers also began growing vegetables on a portion of their land for family consumption as markets became inaccessible or cashflow became limited. Likewise, milk producers managed excess milk production with no means to sell it or alternative sources of disposable income. In a relatively well-off, higher-caste, joint family in UP (S.no. 6, urban, non-migrant, general caste), excess milk production had to be used creatively in the absence of cold storage infrastructure. This included making and consuming a variety of dairy items such as sweets, ghee, butter, and cheese. Even in these varied forms, milk was too much for family and relatives to consume, so a portion was regularly wasted. Meanwhile, since they were unable to sell their milk products, earnings were limited, and they also had difficulty managing day-to-day expenses, such as grains, vegetables, and children’s school fees.

Thus, while these asset-owning higher-caste-class families were affected by disruptions in crop and milk sales, access to land and cattle allowed them to absorb shocks without the need for erosive coping. In these cases, the severity of impact was also cushioned by multiple food sources and multiple income streams. For example, while the elder son in the cattle-owning family lost his job, the household’s food was not dependent on his earnings alone and thus remained food-secure.

However, marginalized households lacking access to assets faced compounded risks arising not only from their caste–class position and limited access to resources and networks, but also from adverse incorporation under neoliberal restructuring of rural economies. These shifts necessitated migration and created multi-sited household vulnerabilities and translocal risks, as livelihoods became dispersed, precarious, and increasingly dependent on unstable market relations. The most telling example of this was changes to crop-sharing arrangements in the context of reverse migration. In some cases, landowners withdrew from *batai* (crop-sharing) arrangements to save costs when their sons returned from cities and could work their own fields, leaving landless laborers without access to work and related grain payments at a time of acute need.

Crop-sharing arrangements in UP include *batai* (one-half), *tihai* (one-third), *chouthai* (one-fourth), and *on theka* (a pre-decided amount of crop must be given to the owner), indicating cost sharing between agricultural laborers and landowners. In some *chouthai* arrangements, laborers contribute their labor and machinery costs but do not share input costs in return for one-fourth of the produce. One of our agricultural laboring participants (S.no. 7, Male, 44, rural, non-migrant, Other Backward Caste) explained that this arrangement was easier for the landowner to break than when laborers also shared a portion of the input costs. His family was severely affected when the landlord broke the arrangement to save costs. He explained that families like his were heavily reliant on this cereal stock for food throughout the year and had limited cash flow to buy it from the market. This case clearly shows how integrated rural economies are now impacted by livelihood insecurity rooted not just in local contexts but also in broader systems and processes. The shock of COVID-19, which triggered reverse migration to rural areas, further strained already struggling rural economies—thereby creating new forms of vulnerability while exacerbating existing ones.

Table 2 summarizes the diverse yet inter-related vulnerabilities of four key groups in our study: Rural non-migrants, circular migrants, urban migrant and urban non-migrants.For both circular and urban migrants, the political economy of rural livelihoods—characterised by limited diversification of farm incomes and limited availability of both farm and non-farm livelihood opportunities—triggered distress migration to urban areas. For urban migrant families, this meant a structural change in their household location and a susbequent break from rural income sources; whereas for the circular migrants, the aim of migration was to secure diversified access to livelihood opportunities for the individual worker, while sometimes retaining seasonal access to rural livelihod opportunities. This seasonal/circular migration of predominantly male workers and longer-term shift to urban areas of families with dependents created complex, multi-sited households that are maintained through affective and financial ties across geographies.

**Table 2:** Experiences of COVID-inter-connections and differences.

Theme	Rural-non-migrant	Circular migrants	Urban-migrant	Urban non-migrant
Access to entitlements (PDS)	Local registrations, doubled rations during COVID	Origin based entitlements, exclusion during mobility	Limited portability/documentation issues, multi-sited households-continued access in origin	Issues of documentation, procedural issues-hampering access
Livelihood opportunities	Small/marginal farmers, agricultural/ contractual labor	Informal work-e.g., daily wage work, food carts	Informal work- e.g., drivers, daily wage work, factory work	Informal work- e.g., painters, construction labor, masons, daily wage work
Political-economy-ties to rural livelihoods	Agrarian distress, lack of diversification, limited non-farm opportunities	Migration as risk diversification to declining rural incomes, ties of affect and care and remittance networks maintained	Migration as survival strategy/social mobility strategy, emotional and financial ties persist- structural shift in location	Competition for limited informal jobs due to rural distress migration
Risk management	Local support networks often tied to class/caste/ religious identities Asset ownership (land/ cattle)-act as buffers	Little/no local support, return to rural origin-reliance on rural absorptive capacity	Access to rural support networks and local social networks if built over time- mediated through caste/religious identities	Local support networks- built through time, work/kin relationships, caste/class/religious networks
Impact of Reverse migration	Disrupted crop-sharing arrangements, stress on limited resources	Return to rural areas-pressures to find local jobs, reliance on family members in rural areas	Parts of multi-sited households, expectations of remittances to support in times of scarcity	Some faced job-loss -like migrants

We find that, while all economically precarious families were at risk in the context of COVID-19, circular migrants were particularly vulnerable. While they migrated to urban areas to mitigate/diversify their existing risks, they generally did not have access to local support networks or standard government entitlements at their destination. This was largely due to their mobile lived realities, documentation issues or family members still accessing their entilements at origin due to which entitlement location was consciously not updated. On the other hand, some rural non-migrant families, mainly small/marginal farmers and agricultural laborers, were heavily reliant on local welfare systems like the PDS, and the additional allocations during lockdowns were especially critical for maintaining food security.

While circular migrants and urban-migrants faced similar disruptions in informal job opportunities, some longer-term urban migrants benefitted from established community based/other social networks in the city, a support that individual circular migrants often lacked. Given the interconnectedness of rural-urban economies, reverse migration increased pressure on limited rural jobs, strained household resources, and intensified dependence on family networks and remittances across all groups. Lastly, while urban non-migrants also faced loss of livelihood opportunities due the disruptions, they too, often had access to local social support networks developed over time that helped them to absrob shocks without the need for erosive coping.

Our findings thus suggest that diverse coping strategies and ability to absorb shocks without resorting to erosive coping, then, depended not only upon access to livelihood opportunities but also local support networks (as embedded in structures of caste-class and length of stay in an area) and access to government entitlements, which acted as important buffers for those with access.

## Discussion

Our study highlights the diversity of experiences with food insecurity during COVID and identifies nuances in access challenges, particularly for precarious households. We find that households adopted a variety of coping strategies when faced with lockdowns, ranging from drastically altered consumption to changes in household structure to making difficult trade-offs between sustenance and investment in future earnings potential.

The availability and effectiveness of these strategies, and their longer-term implications, were situated within intersecting hierarchies of caste, class, gender, and duration of residence. These constituted the foundational conditions through which social networks were formed and mobilised. They shape differential access to social support and thereby compound vulnerabilities for some households while creating relative advantages for others. This also gave rise to multiple in-group/out-group dynamics that shaped access to local support networks and consequently, livelihood opportunities, and determined both the intensity and sustainability of coping. For example, long-term residents in our study had stronger social networks to rely on than circular/new migrants; rural agrarian landed families (often upper castes) had better fallback options for food than rural/urban agricultural/construction labor; women in precarious households were more at risk of nutritional deprivation than other members; and agricultural laborers were more at risk in some crop-sharing arrangements than others.

Importantly, then, our findings suggest that COVID-19 intensified risks associated with exclusion from entitlements and triggered short-term responses such as debt accumulation, but these exacerbating factors were particularly difficult for families to manage because many were already regularly deploying coping strategies in response to longstanding, often intersecting structural inequities.

As evidenced in the literature [8,10,14], some of these coping strategies, which are rooted in structural inequalities, might not be choices at all. Our findings suggest that when households routinely cope with seasonal livelihood stresses and consequent food insecurity, they are chronically operating very close to the resilience threshold. A crisis in such cases can quickly give way to undesirable adaptive strategies or adaptive preferences that are directly at odds with individual well-being and further erode the capacity to cope with future shocks. COVID-era decisions, such as accumulating debt, withdrawing children from school, selling assets, and delaying medical treatment, can impair households’ long-term livelihoods. Thus, an understanding of local support structures, complemented by government entitlements, are essential to contextually situate the sustainability and resilience of individual households.

Policies should thus prioritize timely support for vulnerable livelihood systems in times of crisis to reduce reliance on short-term coping strategies that erode long-term potential and to build more resilient systems.

### Limitations and Strengths

Our study is based on a purposive sample of lower-income households in selected districts of only two states of India— Uttar Pradesh and Goa. The study thus only provides locally contextualized evidence and does not make any generalizations for any group, community, district, state or the country as a whole. While we diversified our sample by caste, religion, household types, and family structures to be inclusive of diverse and marginalized voices, we do not claim to generalize our findings. In addition, it is possible that, despite purposively seeking marginalized households, we missed households facing the greatest precarity. For example, although housing conditions were unsafe, insecure, and unsanitary for some households, these conditions are still not as precarious as having no housing at all.

On the other hand, we use in-depth interviews with multiple members of the same household to provide detailed accounts of the lived realities, meanings associated and mechanisms and support available to diverse groups during a particularly challenging point in time. We foreground participant voices, creating space for marginalized and precarious groups to articulate their realities in their own terms. Qualitative studies like ours thus play a crucial role in deepening understanding by providing evidence of how individuals and households interact with larger socio-economic structures, policies, and hierarchies, thereby contributing to more grounded policymaking. This is particularly relevant to questions of food insecurity, as quantitative studies of nutrition often miss the trade-offs and coping strategies families adopt to address food shortages, which are rooted in socio-cultural and economic structures of inequality that create intersecting vulnerabilities. Additionally, our study makes a unique contribution by interviewing and including the perspectives of older children (7–18-year-olds), who have been labelled a ‘forgotten population’ in the food insecurity literature [18].

Due to practical limitations on fieldwork during COVID, we were unable to conduct interviews during lockdowns and instead relied on retrospective recall, conducting interviews 28 months after the last lockdown (August 2020). While this carries a risk of recall bias, COVID was an extremely memorable, unusual, and disruptive period whose impacts persisted well after the lockdown period. Additionally, even after complete lockdowns were abandoned as a strategy, restrictions on movement and certain types of work remained, affecting livelihoods and incomes. While asking participants to recall experiences after the event carries a risk of recall bias, linking to memorable or emotive events may reduce bias [32–35], particularly when recall focuses on general experiences rather than fine-grained specifics (such as event dates), as was the case in our data. While there is still a risk of recall bias, we note that COVID-19 was a one-of-a-kind, extremely memorable, and emotive period. Lastly, we discuss the ongoing challenges participants link to COVID-19. These include reduced incomes and frequency of workdays and lost businesses, but also long-term effects of shocks such as the death of a breadwinner/support provider or lasting impacts of delaying surgery because of COVID-19. The recollections our participants shared are thus often tied to major upheavals in their lives that were directly caused by the pandemic. Temporal slippage, thus, though a possibility in any study of this nature, might not undermine the discussions as participants relate causality of challenges to the pandemic, even if timelines were blurry.

## Conclusion

Conflicts, disease outbreaks, and global interdependencies that disrupt global supply chains are becoming increasingly common. In this context, it is imperative that governments, communities, and households be better prepared for crisis events such as COVID-19.

While the greater integration of rural economies with national/global labor circuits has created newer forms of livelihood insecurity, it has made migration and multi-sited households a necessary strategy for sustenance in the absence of other forms of support. Our findings indicate that, for households chronically operating close to the resilience threshold, migration reflects not merely a geographic shift but a systemic dispossession, often stripping migrants of vital localized social safety nets (both those provided by government and those built by social networks).

In this context, while social protection schemes like the One Nation One Ration Card (ONORC), which allow migrants to access their PDS allocations from anywhere in the country, offer a vital policy intervention, our findings suggest a lack of policy awareness and structural friction in the transition process. Given that many households were multi-sited, with members residing in multiple jurisdictions, enabling decentralized access to rations based on an individual’s place of residence rather than a single registered household location would better reflect the lived realities of mobile and multi-sited households and individuals. However, our participants also reported challenges with access due to documentation issues, digital barriers, and wage losses from lengthy administrative processes. Building the requisite infrastructure to enable a seamless transfer to ONORC for informal migrant workers, without imposing additional costs, might be crucial to translating an ambitious portability policy into on-the-ground results.

Similarly, addressing knowledge gaps about the policy and reducing technological barriers to access, such as authentication failures and digital divide issues that disproportionately affect manual labor, older people, women, and those with mobility issues, might be critical to ensuring effective implementation. Facilitating easy self-registration on portals like *E-Shram* and leveraging these registrations to streamline national portability of entitlements can also ensure sufficient support is available during disruptions. However, we also note that maintaining non-digital options for accessing entitlements is essential. Extant literature highlights that, because of technology’s long history enforcing systems of marginalization, many reluctant adopters of technology often have *warranted distrust* of technology [36–39]. While addressing *barriers to access* is much-needed, overemphasis on digital registrations risks reducing accessibility of entitlements for users who prefer to opt out of digital technologies.

Lastly, our study reveals that health costs are an important driver of cumulative deprivation in economically precarious households. Thus, broadening the inclusion criteria of schemes like PM-JAY (Health Cards, which allow treatment in the national list of empaneled hospitals, eligibility is determined by Socio-Economic Caste Census) to include circular migrants and informal labor would directly address a major source of resource depletion in precarious households, where limited resources may otherwise be allocated towards addressing concerns of food insecurity.

Thus, our findings contribute to understanding context-specific local social arrangements and practices that create conditions for resilience and adaptability during shocks, and to identifying diverse vulnerabilities to plan for future disruptions. Our study underscores the importance of understanding context-specific household strategies to inform policies that build long-term resilience.

## Supporting information

S1 File Description of the sub-sample. (DOCX)

S2 File Summary of core questions. (DOCX)

S3 File Quantitative summary of themes. (XLSX)

S4 File Inclusivity in global research checklist. (PDF)
