# Supplementary material for: Diverse coping strategies for food insecurity: A qualitative study of economically precarious households in India in the context of COVID-19
Source: PLoS One. 2026 Jun 10;21(6):e0350020. doi: 10.1371/journal.pone.0350020 (PMC13252718; doi:10.1371/journal.pone.0350020)
Supplement: S1 File — (DOCX) [file pone.0350020.s001.docx]

**Details of the households in the sub-sample**

**Kanpur**

***Serial number 1,2,3- Urban non-migrant Joint family with 3 separate nuclear units***

Three families- 2 married brothers and a married sister, along with their mother live together in the same compound in 3 different rooms and together comprise 12 members. They are part of a complex house structure which underwent changes due to two deaths in the family in the lockdown period (and just after) and have been discussed and diagrammatically presented in the paper. While S.no.1 has a small separate kitchen space, s.no. 2 and s.no. 3 both use the space within their rooms to cook. The ventilation in the rooms is limited and there is one dry toilet that all 12 members use. While they operate as separate nuclear families in terms of food and daily finances; they are in-principle a joint family living within the same premises and offer emotional support or childcare when the need arises.

**S.no.1**- This is a 4-member multi-generational-family comprising- the mother (68), her son (37) and two grandchildren (17-year-old girl and 16-year-old boy). The father of the joint family and the son’s wife passed away during Covid. Son, who works as a painter had problems of alcohol abuse even when his wife was alive. However, the problem became extreme after his wife’s death and he is largely an absent parent to the children. Both the children were struggling after the loss of their mother, and their grandmother took up their financial responsibility as well as taking care of their food/ daily needs etc. She works on a makeshift stall by the roadside to earn some money to manage the daily expenses of children. The granddaughter (17) helps by spending time in the stall when her grandmother is home to rest. She also cooks the evening meal for the family, while her grandmother cooks in the morning.

**S.no 2**- has three family members- the disabled daughter (38), her husband (38) and 10-year-old son. The husband is a construction worker and the woman herself sells tobacco, cigarettes and packaged snacks on a makeshift stall by the side of the road.

**S.no.3-** This is the oldest brother’s (43) family who lives with his wife (43) and three unmarried children- 2 daughters aged 17 and 15 and a 10-year-old son. He works as a painter and the daughters also contribute to family income by working as domestic help in a nearby house. Only the boy child is enrolled in school.

**Serial number 4- Seasonally-Migrant rural family from Bihar, workers in a brick kiln**

The family is a seasonal migrant family that spends summers and winters within the premises of the brick-kiln in which they work rural Uttar Pradesh. They are originally from Bihar and work as agricultural/manual labour when in Bihar. They have been seasonally working at various kilns in UP for about 7-8 years now. The kiln is operational during winters and summers when this family and some relatives come and construct a temporary shelter for themselves within the kiln. Food is cooked on earthen chulhas and there is a handpump around which clothes are washed, and temporary cloth structures serve as bathing spaces around that area. During the monsoons, the kiln shuts down for a few months, and the kutcha houses are dismantled/washed away by the rain. More permanent parts like tin roofing (provided by the kiln owner) are securely stored away to be reused upon return before leaving.

This family includes father-mother and their four children, 2 sons(Aged 16 and 14) and 2 daughters (aged 19 and 12). None of the children are married. Older son lives in the village and does not migrate with them anymore, so he has not been considered a part of this household. Two daughters and the younger son, along with their parents all contribute labour to the brick kiln and especially when help is needed to finish the day’s tasks. The younger son was not enrolled in school at the time, and the youngest daughter was enrolled in a civil-society school but was not attending it regularly.

During COVID, the family was living in UP, with little access to public support. They had their PDS registrations in Bihar and relatives, including parents and son were able to access their share during that time, while they relied on non-government support and loans from kiln owners

**Serial number 5- Rural non-migrant joint family with 2 out-migrating brothers**

This is a joint household living in rural UP. Generally, the multi-generational household comprises 8 members, including their mother’s brother (uncle, aged 65); one unmarried sister (43) and one married brother (55), his wife (33) 3 sons (17,13, 12) and a daughter (15). During Covid two more unmarried brothers returned home as they were unable to find work in their usual place of work (a textile factory in a neighbouring state). While one of the unmarried brothers has returned to his usual place of work, during our interviews, one unmarried brother (40) continued to stay here, keeping the family composition to 9 members.

They are a Hindu, dominant caste landless family and the married brother works in a factory. The days of available work are tied to the orders the factory gets. During Covid, the unmarried brothers were also employed in the factory for a limited period. The number of workdays were further reduced by the factory by employing more people to cater to lower orders.They own cattle and sometimes sell milk for extra income. Water is filled from a community hand-pump at some distance from the house. There is a handpump which is nearer to the house, which the family avoids because it used by members of lower caste families. Food is mostly cooked on chulha as gas is too expensive. Women collect and store firewood and make cow-dung cakes. All four children are enrolled in school and attend regularly.

**Serial number 6- Joint family from urban UP, engaged in Milk production and sale**

The family is a higher caste, milk producing family that has an above poverty line ration-card (APL). The father and two married brothers live together along with each of their families. Father (75) was earlier engaged in farming as well, however now they are mostly involved in dairy production and sometimes grow limited crops like wheat and potatoes. The elder brother (50) lives with his wife (45) and 5 children- 2 elder sons (27, 22) and three daughters (19, 17 and 14). While all the children were unmarried, the oldest daughter’s (19) wedding was being planned when we visited. The younger brother (43), his wife (38) and their only daughter (12) also lived in the same compound.

It is a pucca house with 3 rooms and a washroom with water-supply. The family members have constructed the house themselves in the past few years. They generally use a chulha for cooking and LPG rarely, whenever available. All the family expenses were managed from the milk business. Only the youngest girl children (14 and 12) were enrolled in school and the older son of the elder brother was working as a delivery person in the city. All the other family members contributed to the family milk business by managing the cows, preparing fodder and women also manged the household responsibilities. They struggled during Covid as they live with the belief that milk and sons hold the same position of importance and cannot allow for milk to go bad. In the absence of cold supply chain infrastructure and access to markets- not only did earnings from milk suffer, but they also struggled to ensure that milk did not spoil due to heat/lack of storage.

**Serial number 7- Nuclear farming family in rural Uttar Pradesh- son migrated to city to find non-agricultural work**

This is a Hindu, nuclear, backward caste family from rural UP. The husband (44) who works as an agricultural and construction labour, lives with wife (33) and two sons (17, 15) and a daughter (11). While he contributes labour to other’s farm, the mode of involvement changes seasonally or as per availability of labour contracts. For example, when they are involved in crop sharing arrangements like batai/tihai- they get a share of grain in return for their labour at the end of the crop cycle. In these arrangements, if extra labour is hired, those in existing contract must bear its costs. So families involve all members, including children to help-out to complete that tasks at hand and save on labour-hiring costs. These workers also take up additional labour work- which would allow some cash inflow for their families. The children also took-up seasonal jobs like collecting potatoes in the harvest season for a daily wage.

The older son of the family found a job- selling snacks on a cart in a nearby state and had migrated out about 6 months before we conducted the interviews. So, effectively there were 4 members of the family staying in the household. The house was half kutcha and half pucca- with the roof having recently been concretized. The younger son and daughter were both enrolled in school but regularly missed school to contribute labour on the farm where their father was hired.

**Serial number 8-Nuclear farming family in UP, children withdrawn from school**

This is a Hindu, backward caste nuclear family living in rural UP. They own a very small portion of land but also recently started working as agricultural labour. They attach a certain stigma to working on ‘other people’s land’ but agree that it needs to be done to manage sustenance for the family. The husband (42) lives his wife (28) and a daughter (15) and a son (10).

The husband engages in farming activities and also works as agricultural labour, the wife and the daughter contribute to farm activities but are also involved in embroidery work and mange some income from that as well. While both the children were withdrawn from school during and after COVID, only the son had returned to school when the interviews were conducted. Public distribution system served as an important source of food and fallback option for their family.

**Serial number 9- Nuclear, multi-generational rural family work as agricultural labour and own food stalls**

This is a higher-caste Hindu family. The father (60) lives with his son (26), daughter-in-law (23) and their two children (3, 10 months). Pre-Covid, they used to farm their own land. However, their land had to be sold due to health expenses of mother and sister who passed away due to cancer and TB respectively. Post that, they worked as agricultural labour. However, changes in availability of work over the years and decreasing incomes from agriculture, in addition to decreasing capability to engage in hard physical labour forced the father to start a food-cart business. He sells snacks on a cart outside a government college and is supported by the son. During COVID, they faced substantial financial difficulties as they could not find work, and it was difficult to manage everyday expenses and food with a newborn at home. The son took up work as a truck driver during the time to make deliveries and make ends meet.

**Goa**

**Serial number 10 – Second generation migrants- living in Urban Goa**

This is a Hindu, Scheduled Caste family who are second generation migrants- their parents migrated to Goa. The husband’s family is originally from Karnataka and wife’s family is originally from Maharashtra. They have been living in the same locality since a more than 30 years and families have established local connections. They also have local registrations for PDS support.

The family consists of father (73), the married son (37) and his family and an unmarried daughter (40). The son’s wife (35) has multiple chronic illnesses including a heart defect and has two children, a son (8) and a daughter (6). Shortly before our visit, the family had undergone a change in the household structure where the second married brother had separated, and changes were made to the house to facilitate this split. Post this division of space in the home, the father usually ate with the second brother. The unmarried daughter has a mental health condition and needs care. The brother has setup a small shop at their door, selling packaged snacks like biscuits/chips to keep her engaged. However, the family has no earnings from that shop.

The husband(son,37) has a backbone injury and can only do limited work. He works as a driver and the daily expenses of his family and sister are managed solely by his earnings. They faced a difficult time during COVID as he could not get work as colleges were shut and there were no tourists, so he did not receive any cab-hire requests. Both the children are enrolled and attend school regularly.

**Serial number 11- Migrants from Karnataka, support from husband’s parents**

A Hindu Scheduled Caste family, originally from Karnataka, husband (38) migrated to Goa in search of work about 15 years back. The wife moved to Goa to join the husband from Karnataka after marriage. They have 4 children- three daughters (12, 11 and 6) and a son (7). The oldest daughter lives with her paternal grandparents, who are financially better-off, to save costs of travel to her school. She visits home during festivals, weekends and vacation days.

The family lives in a single room dwelling which has a small, segregated space for kitchen/washroom for bathing and use a pay-per-use community toilet. They maintain close connections with relatives in Karnataka and visit at least once a year. They also receive jowar, oil etc., after harvest from maternal relatives, which helps them manage yearly food expenses. The husband has a chronic back injury that needs to be operated upon. He was not working during the time the interviews were held. The wife worked as a domestic help and the family expenses were largely managed through her earnings. During Covid, both the older daughters were sent to live with husband’s parents to save costs and manage the limited resources as livelihood opportunities declined.

**Serial number 12- Migrant multi-sited family from Odisha**

A Hindu nuclear family from Odisha, have been living in Goa for 10 years (approx.), occasionally returning to the village for some months and then coming back gain. At the time of the interview, the husband (42) had returned to the village for rice cultivation on family land. The wife (38) and two children- a son aged 7 and a daughter aged 8- lived in a single room-rented accommodation with a community toilet. Both the children were enrolled in school. Their elder daughter (12) continued to remain in Odisha and was being raised by maternal grandmother. Their PDS allocations, were accessed in Odisha by the relatives and used by the daughter and other family members.

Both the husband and wife worked as daily wage labour in Goa, slowly building local networks for finding work over the years.

**Serial number 13- Family from Madhya Pradesh, child born during COVID**

This is a Hindu nuclear family who is originally from Madhya Pradesh and have been living in Goa for about 15 years. While they have strong emotional connections with relatives in Madhya Pradesh, they do not receive any direct financial/material help from relatives in MP. The family consists of husband (46) wife (38) and 4 sons (21, 18, 17 and 2). The youngest son was born during the COVID period. While the husband works as a tailor, the older son is undergoing a technical training and hopes to get a job in the sector. The 18-year-old assists with a local catering business and manages to get some earning home, However, neither of the incomes are steady and it often becomes difficult to make ends meet.

**Serial number 14- Goan Christian family – single mother with 4 sons**

The family consists of mother (44) and her 3 sons (16,11,9). She works at a temporary food cart near a hotel in the evenings selling Goan snacks. She also has some secondary earnings from cab hire services and selling milk when possible. Her husband passed away in an accident about 9 years ago.

The house she lives in is owned by the brother-in-law who stays in a separate portion of the house. All the three children are enrolled and attend school. They receive rice from PDS allocations which the mother cooks for children. She has specific health conditions and eats boiled/plain food only. They were supported by a cousin, whose family lived in the UK. During the time of our interviews, he had recently left for the UK, leaving the mother tense and emotional.

**Serial number 15 –Second-generation migrant Muslim family from Karnataka**

This family comprises a 36-year-old female, who works as domestic help and her three children- a girl child aged 14 and 2 boys aged 7 and 4 respectively. Her husband owned a food business which shut down due to COVID-19 and he left to an unknown location to avoid debtors. This left the woman in an especially precarious situation. The landlord forced her and her three children to vacate the place when they could not manage rent during the period. She finally found a place to stay and started working as a domestic help to support the children. While the father was not in touch with them for 2 years during the Covid period, he had established contact again by the time of the interviews. The family reported that he called sometimes. However, they received no financial support from him.

| **S.no** | **State** | **Location** | **Government Caste Category** | **Religion** | **Household type** | **Family-type** | **Migration Status** |
| --- | --- | --- | --- | --- | --- | --- | --- |
| 1 | UP | Rural | Data not enough | Hindu | Single | Nuclear | Seasonal migrant family |
| 2 | UP | Urban | Scheduled Caste | Hindu | Joint | Nuclear | Non-Migrant |
| 3 | UP | Urban | Scheduled Caste | Hindu | Joint | Multi-generational | Non-Migrant |
| 4 | UP | Urban | Scheduled Caste | Hindu | Joint | Nuclear | Non-Migrant |
| 5 | UP | Rural | General | Hindu | Joint | Multi-generational | Migrant members |
| 6 | UP | Urban | General | Hindu | Joint | Multi-generational | Non-Migrant |
| 7 | UP | Rural | Other Backward Caste | Hindu | Single | Nuclear | Migrant members |
| 8 | UP | Rural | Other Backward Caste | Hindu | Single | Nuclear | Non-Migrant |
| 9 | UP | Rural | General | Hindu | Single | Multi-generational | Non-Migrant |
| 10 | Goa | Urban | Scheduled Caste | Hindu | Joint | Multi-generational | Migrant family (second generation) |
| 11 | Goa | Urban | Scheduled Caste | Hindu | Single | Nuclear | Migrant family |
| 12 | Goa | Urban | Data not enough | Hindu | Single | Nuclear | Migrant family |
| 13 | Goa | Urban | Data not enough | Hindu | Single | Nuclear | Migrant family |
| 14 | Goa | Urban | Undeclared | Christian | Single | Single mother | Non-Migrant |
| 15 | Goa | Rural | Undeclared | Muslim | Single | Nuclear | Migrant family (second generation) |
