# Supplementary material for: Diverse coping strategies for food insecurity: A qualitative study of economically precarious households in India in the context of COVID-19
Source: PLoS One. 2026 Jun 10;21(6):e0350020. doi: 10.1371/journal.pone.0350020 (PMC13252718; doi:10.1371/journal.pone.0350020)
Supplement: S2 File — (DOCX) [file pone.0350020.s002.docx]

**Summary of Core questions**

1. **Household roster**

This instrument was completed by one adult representative of the household to get a preliminary sense of the socio-demographic characteristics of the household. The roster began with questions on housing characteristics to gauge the living conditions. These included questions around ownership status of the house/monthly rent, the presence of a separate kitchen and/or toilet facilities, main source of water and cooking fuel. We also collected information about the household income, main earner, caste, religion and the type of ration card. After household-level questions which were common to all members, we collected person-specific information, including each household member’s name, age, gender, relationship to the household member filling the roster, primary work (if applicable), previous work (if any) and years and type of education. Lastly, we collected details of members who had left the household within the last 12 months.

1. **Children’s interview-guides (Ages 7-18)**

While questions for 7-12 year olds were asked using simplified language, and children under age 13 were offered the option to draw if they wished, the content of the instruments was otherwise identical for children aged 7-12 and aged 13+. After reassuring the children that there were no right or wrong answers and we only wanted to understand their experiences, we started with questions about meals at school, including if lunch was provided, who they ate/did not eat with, foods they enjoyed and if they preferred home-cooked food or meals at school.

If the children were not attending school, we started with questions about their daily routines and probes related to meals. We also asked questions about their favourite foods, foods they did not like, and foods that they wanted to eat but did not have access to, to understand their food environments. We also asked specific questions around meal preparation and mealtimes like who cooks, who serves, who eats first/last and description and probes about the time when everyone sits together and eats etc. COVID-specific questions included- do you remember the pandemic, was there a time when the schools were closed, did COVID make it harder to access food and who helped the family during that period. In addition, we administered a child-specific food insecurity experience scale which captures food insecurity-related experiences such as about being worried about food or having had to skip meals, feeling weak due to not eating or going hungry the entire day.

To allow children to dissociate and answer, we also used vignettes to explore their understanding of food scarcity and limited food diversity by giving them two case studies of fictional children experiencing some food insecurity and asking them to guess the reasons for such experiences. Lastly, we also asked children about their responsibilities at home and if they had ever engaged in paid work.

1. **Adult interview guides (18+)**

Adult interview guides were very detailed. We began with questions on migration status to understand how long a family had been in a particular place, if they had recently changed their occupation and if/how the transition had affected the availability of food or their health and well-being more generally.

After this, we asked questions about the infrastructure of the house, including if they had any repair or construction done recently, what the source of water was for the household and if there were any challenges in access to water. Next, we asked questions regarding cooking, starting with major source of cooking fuel, preferred cooking fuel and related issues of access. We explored food sources and responsibilities at home through questions like who buys groceries, who cooks, from where do you buy food/groceries etc. Lastly, we asked questions around ideas around nutrition and preferred foods, important foods for children to consume, what is nutritious and what was not healthy etc. We administered the UN Food and Agriculture’s Food Insecurity Experience Scale (FIES) for , followed by qualitative probes to understand instances of food insecurity more deeply. COVID-specific questions included whether/how COVID-19 affected the household’s ability to get enough food including issues related to affordability, access, supply and preparation in the household and the kinds of government support that was available. Even though the recall period for FIES is last 12 months, many of FIES responses generated discussions around COVID-19 and incidents that happened in the context of lockdowns.
